# Clinical characteristics of cerebral vascular dementia and early diagnostic value of cranial nuclear magnetic resonance

**DOI:** 10.12669/pjms.39.2.6425

**Published:** 2023

**Authors:** Wenxing Zhou, Lianxia Li, Yan Lang, Hongxin Jiang

**Affiliations:** 1 Wenxing Zhou, Department of Radiology, Gucheng County Hospital, Hebei Province, 253800, China; 2 Lianxia Li, Department of Radiology, Gucheng County Hospital, Hebei Province, 253800, China; 3 Yan Lang, Department of Radiology, Gucheng County Hospital, Hebei Province, 253800, China; 4 Hongxin Jiang, Department of Radiology, Gucheng County Hospital, Hebei Province, 253800, China

**Keywords:** Cranial magnetic resonance imaging, Ischemic stroke, Vascular dementia, Hippocampal volume

## Abstract

**Objective:**

This study aimed to observe the diagnostic value of cranial magnetic resonance imaging (MRI) in patients with vascular dementia induced by ischemic stroke.

**Methods:**

The experiment was designed according to the randomized control principle. Two hundred and eighty patients with ischemic stroke who were admitted to Gucheng County Hospital between June 2019 and June 2021 were selected as research subjects. Patients without vascular dementia after stroke were included in the control group, and patients with vascular dementia after stroke were included in the observation group. The cranial MRI was performed in both groups.

**Result:**

Proportions of patients with large and moderate infarct lesions in brain tissues were significantly higher in the observation group than the control group. The data variation of relevant MRI detection indicators of the observation group was more obvious than that of the control group (P<0.05). The mini-mental state examination (MMSE) and Montreal cognitive assessment (MoCA) scores of the observation group were lower than those of the control group (P<0.05), but the HIS score was higher (P>0.05). Patients with changes in brain morphology were more in the observation group than the control group (P<0.05).

**Conclusion:**

Patients with vascular dementia induced by ischemic stroke are characterized by cortical atrophy, widening of the cerebral sulcus, large infarct lesion area and sparse cerebral white matter. Cranial MRI can effectively identify these features. The application of cranial MRI has some clinical values for early treatment and prognostic assessment.

## INTRODUCTION

Vascular dementia refers to a syndrome of intellectual and cognitive impairment caused by cerebrovascular diseases such as ischemic stroke and hemorrhagic stroke.^1^,^2^ A study has shown that vascular dementia is an acquired disease,^3^ its incidence is closely related to ageing, the prevalence of vascular dementia in people over 65 years of age in China is about 1.5%, and vascular dementia cases account for 20% to 30% of all dementia cases. Since the pathogenesis and pathological basis of vascular dementia are relatively special, most patients often have different underlying cerebrovascular diseases, and ischemic stroke is the most common.^4^,^5^ The risk of cerebrovascular lesions increases substantially in the elderly population due to the reduced metabolic capacity of peripheral circulation and vascular smooth muscle motor regulation. Once cerebrovascular lesions occur, blood circulation is often impaired, and the local brain tissue is continuously undersupplied with blood and oxygen. With the prolongation of the disease, the degree of brain tissue damage intensifies, leading to slowly progressive cognitive dysfunction and eventually to the development of vascular dementia, which has a serious negative impact on the quality of life and imposes a heavy economic and psychological burden on patients’ families. Unlike other dementia diseases with complex etiology and unclear pathogenesis such as Alzheimer’s disease, vascular dementia has clear causative factors and progresses slowly, so early diagnosis and targeted treatment can effectively improve the prognosis of patients and their quality of life.

Evaluating the degree of cognitive impairment based on medical history and neurocognitive scale is the main way to diagnose vascular dementia at this stage of clinical practice. However, the diagnostic method is greatly interfered with subjective factors, and the complex diagnostic process and poor quantitative follow-up diagnostic efficacy make it difficult to confirm the occurrence order of cerebrovascular lesions and cognitive impairment in terms of time. Moreover, the hidden progress of most patients makes it impossible to monitor the progress of patients timely and effectively. In recent years, with the development of medical imaging technology, the use of neuroimaging is currently the main diagnostic means of vascular dementia. computed tomography (CT) and magnetic resonance imaging (MRI) are common neuroimaging means, and CT that shows more significant advantages have been gradually applied in the clinical diagnosis and treatment of various brain tissue lesions.^6^-^8^ This paper analyzed the characteristics of vascular dementia induced by ischemic stroke and the advantages of using cranial MRI for early diagnosis and treatment, with the aim of providing a reference for early diagnosis and treatment of this disease and providing clinical lessons.

## METHODS

The experiment was designed according to the randomized control principle. Two hundred and eighty ischemic stroke patients admitted to our hospital from June 2019 to June 2021 were selected for the study. Patients who did not develop vascular dementia after stroke were included in the control group (205 cases), and patients who developed vascular dementia after stroke were included in the observation group (75 cases). In the control group, there were 114 males and 91 females, with ages ranging from 60 to 83 years (average (72.4±3.1) years). As to the education level, 20 patients graduated from colleges or above, 49 patients graduated from high school, and 136 patients graduated from junior high school and below. In the observation group, there were 41 males and 34 females, with ages ranging from 61 to 84 years (average (73.5±3.2) years). As to the education level, six patients graduated from colleges or above, 16 patients graduated from high school, and 136 patients graduated from junior high school or below. There were no statistically significant differences in the general data including gender, age and education level between the two groups (P>0.05); thus, the results were comparable. All patients and their families clearly understood the content and purpose of the study and signed the informed consent. The study protocol was reviewed and approved by the ethics committee of the hospital (Approval No. 202104260019 dated on April 26^th^ 2021).

The diagnosis of ischemic stroke followed the criteria in *Guidelines for the Diagnosis and* Treatment of Acute Ischemic Stroke in China (2010) released by Neurology Branch of Chinese Medical Association in 2010.^9^ The diagnosis of vascular dementia followed the criteria in Draft of Diagnostic Criteria for Vascular Dementia released by Neurology Branch of Chinese Medical Association in 2002.^10^

### Inclusion criteria:

- Meeting the diagnostic criteria of ischemic stroke and vascular dementia;
- Having no relevant cognitive impairment before the stroke;
- Aging 40-81 years;
- Showing at least one ischemic lesion under cranial MRI;
- Mini-mental state examination (MMSE) score ≤ 24 points, hachinski incheinic score (HIS) ≥ 7 points, Montreal Cognitive Assessment (MoCA) score < 26 points.

### Exclusion criteria:

- Having dementia caused by other diseases;
- Having Alzheimer’s disease;
- Combined with other diseases affecting cognitive function, such as Parkinson’s disease, intracranial tumors, traumatic brain injury, encephalitis, etc.;
- Having psychiatric and psychological diseases.

### Research methodology:

All subjects were evaluated by MMSE, HIS, and MoCA to diagnose vascular dementia and examined by cranial MRI using a 1.5T SignaHDxt MRI system (GR company, USA) one month after acute ischemic stroke. All the patients took a supine position, and the diagnostic operations for all the patients were the same. The patients were first scanned with three sequences, T1W1, T2W2 and Flair, and then with 3D-FSPGR. The scanning parameters were as follows: TR was 10 ms, TI was 300 ms, TE was 4 ms, the scanning field was set as 25 cm × 25 cm, and the flip angle was set as 120°, 116 scanning images were acquired, and the thickness of each image layer was set as 1.6 mm.

### Observed indicators:

The infarct lesions in the brain were observed. The infarcts were classified according to the size of the lesions, including large infarcts: lesions involving more than one lobe, a diameter > 5.0 cm; medium infarcts: lesions involving less than one lobe, a diameter between 3.0 cm and 5.0 cm; small infarcts: lesions involving less than one lobe, a diameter between 1.5 cm and 2.9 cm; lacunar infarcts: lesions involving less than one lobe, a diameter < 1.5 cm. The cortical MRI indexes, such as left-to-right hippocampal volumes, temporal lobe uncus spacing and left-to-right cerebral diameter, were measured. The mini-mental state examination (MMSE), Harchinski ischemic scale (HIS) and Montreal cognitive assessment (MoCA) scores were measured. Two experts with a professional title of attending doctor or above scored the patients according to the items in relevant scales. They also observed the morphological changes of the brain tissue under MRI to evaluate whether there was cortical atrophy, cerebral sulcus widening, widening of the medial hippocampal cerebrospinal fluid pool and leukoaraiosis.

### Statistical analysis:

The data obtained were analyzed and studied using SPSS 22.0. The measurement data were expressed by (Mean±SD) and processed by t-test. The count data were expressed by [n (%)] and processed by Chi-square test. The difference was considered statistically significant when P<0.05.

## RESULTS

The comparison between the MMSE, HIS, and MoCA scores between the two groups suggested that the MMSE and MoCA scores of the observation group were lower than those of the control group (P<0.05), and the HIS score was higher in the observation group than in the control group (P<0.05, Table-I).

**Table-I:** MMSE, HIS and MoCA scores between the two groups (*χ̅*±*s*, point).

Group	Observation group	Control group	t	P
MMSE	15.31±1.88	19.26±3.38	6.327	<0.001
HIS	11.82±3.27	6.06±1.82	9.818	<0.001
MoCA	16.08±3.01	21.86±2.18	9.966	<0.001

The proportion of patients with large and medium infarct lesions in the observation group was significantly higher than that in the control group (all P<0.05). The differences in the proportions of patients with small and lacunar infarct lesions between the two groups were not statistically significant (all P>0.05, Table II).

**Table-II:** Size of infarct lesions in brain tissue under MRI in two groups [n(%)].

Group	Observation group	Control group	X^2^	P
Large infarcts	38(50.67)	45(21.95)	15.927	<0.001
Medium infarcts	42(56.00)	49(23.90)	19.098	<0.001
Small infarcts	56(74.67)	156(76.10)	0.146	0.707
Lacunar infarcts	47(62.67)	135(65.85)	0.268	0.625

The left and right hippocampal volumes and the left-to-right cerebral diameter were significantly smaller in the observation group than in the control group. The temporal lobe uncus spacing was significantly larger in the observation group than in the control group (all P<0.05, Table-III).

**Table-III:** Hippocampal volume, temporal lobe uncus spacing and left-to-right cerebral diameter in the two groups (*χ̅*±*s*).

Group	Observation group	Control group	t	P
Left hippocampal volume (cm^3^)	2.23±0.19	2.57±0.22	10.476	<0.001
Right hippocampal volume (cm^3^)	2.25±0.18	2.60±0.26	9.531	<0.001
Temporal lobe uncus (mm)	29.52±3.23	25.63±3.06	7.780	<0.001
Left-to-right cerebral diameter (mm)	131.48±5.45	136.81±5.80	5.283	<0.001

The comparison of the brain tissue morphology between the two groups found that patients with cortical atrophy, cerebral sulcus widening, widening of the medial hippocampal cerebrospinal fluid pool and leukoaraiosis were more in the observation group than in the control group (P<0.05, Table-IV).

**Table-IV:** Morphological changes of the brain tissue under MRI between the two groups [n(%)].

Group	Observation group	Control group	t	P
Cortical atrophy	38(50.67)	29(14.15)	11.843	<0.001
Cerebral sulcus widening	39(52.00)	24(11.71)	15.419	0.000
Widening of the medial hippocampal cerebrospinal fluid pool	34(45.33)	15(7.32)	15.036	0.000
Leukoaraiosis	63(84.00)	34(16.59)	36.172	0.000

## DISCUSSION

With the development of imaging technology, its application in the medical field continues to broaden. Like other neurological conditions, cognitive disorders can be evaluated early using structural imaging methods. Relevant studies have shown that objective morphological changes, mainly manifested by ventricular widening, vascular damage, or subcortical nucleus degeneration, are often present in cognitive disorders,^11^,^12^ which provides a basis for the early diagnosis of cognitive disorders. The results of a study carried out by Qin showed that changes in MRI characteristic parameters such as hippocampal volume, temporal lobe sulcus spacing, and left-to-right brain diameter could effectively distinguish vascular dementia from Alzheimer’s disease, and cranial MRI played a positive role in the differential diagnosis of the two diseases.^13^ In summary, cranial MRI is mostly applied clinically to distinguish vascular dementia from Alzheimer’s disease;^14^,^15^ however, the MRI characteristics of vascular dementia induced by stroke have rarely been summarized systematically, so the experimental results of this study fill the gap in this field.

In this study, the MRI examination found that the proportions of patients with large and medium infarct lesions in the observation group were significantly higher than those in the control group, indicating that patients with vascular dementia induced by ischemic stroke had more severe stroke infarcts and patients with larger infarct lesions were at higher risk of developing vascular dementia. The left and right hippocampal volumes and the left-to-right brain diameter of patients in the observation group were significantly smaller than those in the control group, while the temporal lobe uncus spacing of patients in the observation group was significantly larger than that in the control group, indicating that the degree of cortical atrophy was more severe in patients with vascular dementia induced by ischemic stroke than in patients without vascular dementia. A study by Gu et al. showed that patients with vascular dementia induced by ischemic stroke had cortical atrophy,^16^ widening of the sulcus, and larger infarct lesions under MRI, which is consistent with the finding of this study. It has also been found that the presence of lacunar cerebral infarction, cerebral white matter hyperintensities, microscopic intracerebral hemorrhagic foci, and cerebral atrophy in the brain of vascular dementia patients can be detected by CT and MRI, along with pathological changes such as widening of the cerebral sulcus and cerebral fissure and infarct lesions in key areas of the brain,^17^ which provides a reference for the diagnosis of vascular dementia. In this study, ischemic stroke patients with vascular dementia showed cortical atrophy, sulcus widening, widening of the medial hippocampal pool, and vascular dementia under MRI, and the proportion was significantly higher than that in the control group, which was consistent with the research results as mentioned earlier. Widening of the medial hippocampal pool indicates hippocampal atrophy. The hippocampus is an important area of the brain responsible for memory functions, and hippocampal tissue damages exacerbate cognitive impairment. This indicates that patients with ischemic stroke combined with vascular dementia have altered cognitive function, but more importantly, the altered cognitive function may be caused by changes in brain tissues, which needs further discussion.

The MMSE scale can comprehensively, accurately, and rapidly reflect the subjects’ intellectual status and the degree of cognitive deficits to provide a scientific basis for clinical psychological diagnosis and treatment.^18^ This study found that the MMSE scores of vascular dementia patients were lower than those of the control group, suggesting that their cognitive functions were impaired. The HIS is a simple screening scale for vascular dementia developed by Hachinski in 1975, specifically for the simple screening and identification of vascular dementia, and a HIS score higher than seven points is evaluated as vascular dementia.^19^ The HIS score of the vascular dementia patients in this study was significantly higher than seven points, which is consistent with the results of the above study. The MoCA scale is used as a rapid screening tool for cognitive impairment, including attention and concentration, executive function, memory, language, visual-spatial structural skills, and abstract thinking, covering a wide range of cognitive domains than the MMSE. The lower the MoCA score is, the poorer the cognitive impairment is. The MoCA scale is an extensively applied cognitive function screening scale in clinics.^20^ In this study, the MoCA score of the patients with vascular dementia was lower than that of the control group, indicating that patients with vascular dementia had cognitive impairment. This was consistent with the MRI manifestations. Most of the previous studies only discussed the results of imaging tests, while this paper deepens the reliability of the experimental results by testing the scales mentioned above, which is one of the innovations of this study.

### Limitations:

This study is single-center, so the results were inevitably biased; a multicenter study will be conducted in the future to enhance the reliability of the findings.

## CONCLUSION

Cortical atrophy, sulcus widening, and infarct lesions at different sites can be seen on the MRI images of patients with ischemic stroke-induced vascular dementia, and cranial MRI can effectively diagnose vascular dementia induced by ischemic stroke. Cranial MRI has clinical values for early treatment and prognosis assessment of vascular dementia induced by ischemic stroke.

### Authors’ Contribution:

**WXZ & LXL:** Study design, data collection and analysis.

**LXL & YL:** Manuscript preparation, drafting and revising.

**WXZ & HXJ:** Review and final approval of manuscript; responsible and accountable for the accuracy or integrity of the work.
